# Module-level recombination drives *DBLMSP* polymorphism and functional conservation in *Plasmodium falciparum*

**DOI:** 10.1051/parasite/2026024

**Published:** 2026-04-17

**Authors:** Yi-Wen Duan, Shen-Bo Chen, Tian-Yu Wang, Wan-Xuan Yang, Kokouvi Kassegne, Hai-Mo Shen, Jun-Hu Chen

**Affiliations:** 1 National Institute of Parasitic Diseases, Chinese Center for Diseases Control and Prevention (Chinese Center for Tropical Diseases Research), National Key Laboratory of Intelligent Tracking and Forecasting for Infectious Diseases, NHC Key Laboratory of Parasite and Vector Biology; WHO Collaborating Centre for Tropical Diseases; National Centre for International Research on Tropical Diseases Shanghai 200025 PR China; 2 School of Global Health, Chinese Centre for Tropical Diseases Research, Shanghai Jiao Tong University School of Medicine Shanghai 200025 PR China

**Keywords:** *Plasmodium falciparum*, *DBLMSP*, Modular polymorphism, Antigenic diversity, Intertypic recombination

## Abstract

The *DBLMSP* gene family in *Plasmodium falciparum* encodes surface antigens involved in immune evasion and red blood cell invasion, yet its extensive polymorphism has long defied straightforward classification. While analyzing *DBLMSP1* sequences from samples collected along the China–Myanmar border, we found that haplotypes could not be readily explained by standard population genetic models. Instead, comparative alignment and BLAST analysis revealed that *DBLMSP1* and *DBLMSP2* consist of discrete, recombinable sequence modules, flanked by conserved upstream and downstream regions. This led us to propose a modular framework that redefines allele structure as combinations of well-defined building blocks with consistent boundaries and positional constraints. Through global mining of *DBLMSP* sequences, we identified nine genotypes each for *DBLMSP1* and *DBLMSP2*, with modules labeled sequentially (*e.g.*, 1M2a, 1M3c, and 2M3b). Some modules were shared across paralogs, notably the identical sequence of *DBLMSP1* module 1M3c and *DBLMSP2* module 2M3a, suggesting historical inter-locus recombination. In the dominant genotype DBLMSP1-1, nucleotide diversity and Tajima’s D peaked within variable modules, whereas conserved structural elements, including the receptor-binding cleft and SPAM domain, were under purifying selection. Patterns of long-range linkage disequilibrium aligned with module junctions, suggesting that modular structure may shape recombination patterns independently of selection. Modular recombination has been widely recognized in viral systems and multigene families such as *var*, but its relevance in *DBLMSP*s has been underappreciated. By applying this framework to *P. falciparum DBLMSP*s, we aim to provide a useful perspective for understanding their structural diversity and evolutionary dynamics, with implications for immunogen design and parasite surveillance.

## Abstract (fr)

La famille de gènes *DBLMSP* chez *Plasmodium falciparum* code des antigènes de surface impliqués dans l’échappement immunitaire et l’invasion des globules rouges. Cependant, son polymorphisme étendu a longtemps résisté à une classification simple. L’analyse des séquences *DBLMSP1* d’échantillons prélevés le long de la frontière sino-birmane a révélé que les haplotypes ne pouvaient être facilement expliqués par les modèles de génétique des populations classiques. En revanche, l’alignement comparatif et l’analyse BLAST ont montré que *DBLMSP1* et *DBLMSP2* sont constitués de modules de séquences discrets et recombinables, flanqués de régions conservées en amont et en aval. Ceci nous a conduits à proposer un cadre modulaire qui redéfinit la structure allélique comme une combinaison d’éléments constitutifs bien définis, présentant des limites et des contraintes positionnelles cohérentes. Grâce à l’analyse globale des séquences DBLMSP, nous avons identifié neuf génotypes pour *DBLMSP1* et neuf pour *DBLMSP2*, avec des modules numérotés séquentiellement (par exemple, 1M2a, 1M3c, 2M3b). Certains modules étaient partagés entre les paralogues, notamment la séquence identique des modules 1M3c de *DBLMSP1* et 2M3a de *DBLMSP2*, suggérant une recombinaison inter-locus ancienne. Dans le génotype dominant *DBLMSP1-1*, la diversité nucléotidique et le D de Tajima atteignaient leur maximum au sein des modules variables, tandis que les éléments structuraux conservés, tels que le site de liaison au récepteur et le domaine SPAM, étaient soumis à une sélection purificatrice. Les profils de déséquilibre de liaison à longue distance s’alignaient avec les jonctions des modules, suggérant que la structure modulaire pourrait influencer les profils de recombinaison indépendamment de la sélection. La recombinaison modulaire est largement reconnue dans les systèmes viraux et les familles multigéniques comme *var*, mais son importance dans les *DBLMSP* a été sous-estimée. En appliquant ce cadre aux *DBLMSP* de *P. falciparum*, nous visons à fournir une perspective utile pour comprendre leur diversité structurelle et leur dynamique évolutive, avec des implications pour la conception d’immunogènes et la surveillance des parasites.

## Introduction

*Plasmodium falciparum* is the most lethal human malaria parasite, responsible for over 597,000 deaths annually worldwide [45], with the highest burden in sub-Saharan Africa and parts of Southeast Asia [36]. Although China was certified malaria-free by the World Health Organization in 2021 [11], imported cases continue to occur, particularly in border regions such as the China–Myanmar border (CMB), which remains a hotspot for *P. falciparum* reintroduction [40].

Merozoite surface proteins (MSPs) are central to the parasite’s ability to invade red blood cells and are major targets of naturally acquired immunity [4]. Among these, *DBLMSP1* and *DBLMSP2* stand out for their remarkable polymorphism [7, 31]. These proteins contain a Duffy binding-like (DBL) domain enriched with low-complexity repeats, as well as a conserved C-terminal SPAM domain, similar to those found in MSP3 family members [17, 22, 44]. In addition to mediating erythrocyte adhesion, they have been shown to bind host IgM, potentially masking the parasite from immune detection, and they elicit robust antibody responses. *DBLMSP1* (PF3D7_1035700) and *DBLMSP2* (PF3D7_1036300) are single-copy genes located within an eight-member MSP3-like gene cluster on chromosome 10, separated by several kilobases and intervening paralogs [42]. Both genes consist of two exons interrupted by a single intron, and encode large, cysteine-rich proteins featuring an N-terminal DBL domain and a C-terminal SPAM domain [47]. Comparative genomic analyses suggest that *DBLMSP1* and *DBLMSP2* evolved via gene duplication followed by interlocus gene conversion, particularly in the DBL domain region, resulting in mosaic haplotypes and shared sequence blocks between the two genes.

Despite these functional parallels, the nomenclature and classification of *DBLMSP1* and *DBLMSP2* remain inconsistent in the literature. In 2012, Hodder et al. proposed the names *PfMSPDBL1* and *PfMSPDBL2* based on SPAM domain presence in PF10_0348 and PF10_0355 [24]. Later, in 2019, Böhme *et al.* formally annotated these genes as *DBLMSP* (PF3D7_1035700) and *DBLMSP2* (PF3D7_1036300) in PlasmoDB [2, 5, 19]. However, we believe that this binary classification may not fully account for the extensive allelic and structural variation observed in natural isolates. In fact, many sequences differ dramatically in length and internal composition, and cannot be cleanly assigned to either category.

Most prior studies have approached *DBLMSP1/2* from a population genetic perspective, often treating each gene as a single, indivisible unit [14, 30]. However, we believe these antigens may have an internal modular structure. The DBL domains that are generally considered to be functionally cohesive may actually be mosaic structures composed of smaller, recombinable sequence blocks. This possibility has not been explored in detail. Our investigation began with *DBLMSP1* sequencing of *P. falciparum* isolates from the CMB region, a key entry point for imported malaria cases in China [12]. We initially intended to perform standard population genetic analysis. However, early alignment and BLAST searches revealed that many haplotypes did not differ in the typical ways, by random point mutations, but appeared to be formed from recurring combinations of distinct sequence fragments. These fragments had consistent positions and well-defined boundaries, which led us to suspect the existence of a modular organization within *DBLMSP*s.

As we explored further through sequence mining and literature review, it became increasingly clear that existing nomenclature systems fall short in representing this modular pattern. We therefore propose a redefinition of *DBLMSP1* and *DBLMSP2* gene structure based on modular intertypic homologous recombination [35], a mechanism well-documented in viral genomes [21, 46, 48], and increasingly discussed in other eukaryotic systems, but not widely characterized in malaria parasites. Through global sequence analysis and manual segmentation, we identified 18 genotypes, nine each for *DBLMSP1* and *DBLMSP2*, based on reproducible combinations of modules (*e.g.*, 1M2a, 1M3c, and 2M1b). While this framework is still exploratory, we hope it offers a more biologically grounded way to interpret antigenic variation, preserve functional cores, and reconsider how immune evasion may be orchestrated in *Plasmodium* surface proteins.

## Methods

### Ethics approval and consent to participate

The study was conducted in accordance with the principles of the Declaration of Helsinki. Before blood collection, the study protocol and potential risks and benefits were explained to the participants, and written informed consent was obtained from all adult participants and parents or legal guardians of children. Blood samples were collected following the institutional ethical guidelines reviewed and approved by the Ethics Committee of the National Institute of Parasitic Diseases, Chinese Center for Disease Control and Prevention.

### Sample collection and PCR amplification

Blood samples were collected from patients infected with *P. falciparum* in the CMB region. All samples were microscopically confirmed and validated as *P. falciparum* single infections via nested PCR. The *DBLMSP1* gene (PF3D7_1035700) from the 3D7 reference strain was targeted for amplification. Specific primers were designed and synthesized by Shanghai Yingjun Biotechnology Co., Ltd. (Forward: 5′–CACATTTAATTAAGGTTGTATTTAC–3′; Reverse: 5′–ATGTGAAAGCATATATTAAGAACAA–3′).

PCR was conducted using PrimeSTAR GXL DNA Polymerase (TaKaRa) in a total reaction volume of 25.0 μL. The reaction mixture contained 5.0 μL of 5× PrimeSTAR GXL Buffer, 2.0 μL of dNTP Mixture (2.5 mM each), 1.0 μL each of forward and reverse primers (10 μM), 3.0 μL of genomic DNA template, 0.5 μL of PrimeSTAR GXL DNA Polymerase, and 12.5 μL of nuclease-free water. The thermal cycling protocol was as follows: initial denaturation at 98 °C for 3 min; 35 cycles of denaturation at 98 °C for 10 s, annealing at 55 °C for 15 sec, and extension at 68 °C for 3 min; followed by a final extension at 68 °C for 10 min. Amplified products were sent to BGI (Beijing Genomics Institute, Shanghai, China) for bidirectional Sanger sequencing. The 3D7 reference genome was used for sequence annotation and comparative analysis. We note that Sanger sequencing preferentially reflects the dominant allele present in a mixed infection or polyclonal template. Thus, our analysis is biased toward the most abundant haplotypes and may underestimate low-frequency variants present in the same sample. However, our focus in this study was on modular patterns reconstructed across high-confidence sequences; as such, we do not believe this limitation materially affects our conclusions regarding modular structure or recombination boundaries.

### Sequence retrieval and alignment

We selected three representative sequences from the CMB dataset: CMB10 (type 1), CMB42 (type 2), and CMB20 (type 3), representing three different haplotype groups, and performed a blastn search. [10]. The blast results are summarized in Tables S1–S3. Sequence alignment was performed using the MEGA6 and MUSCLE algorithms [43]. We additionally used a conserved 5′ fragment (1CM_up) as a BLAST query to validate the conservation of flanking regions. The returned sequences showed consistent segment boundaries with our proposed modular divisions (Table S4). We then conducted BLAST searches using each candidate module (*e.g.*, 1M2a, 1M2b, 1M3a, *etc.*) as an independent query. The results demonstrated that these modules are polymorphic, *i.e.*, distinct sequence variants occupy the same genomic positions across different genotypes. Representative sequences from each module variant group (*e.g.*, 2a, 2b, 3a, 3b, and 3c) were selected and are highlighted in bold in Supplementary Tables S5–S9.

We found one module (1M3c) yielding high-scoring hits to *DBLMSP1* and *DBLMSP2* (PF3D7_1036300) at the same time, which prompting an expanded analysis of *DBLMSP2*. Removing *DBLMSP1* hits from the 1M3c BLAST output allowed us to extract a conserved 5′ region specific to *DBLMSP2*, which was then used as a new BLAST query to identify diverse *DBLMSP2* homologs. These results revealed that *DBLMSP2* also exhibits a modular structure analogous to *DBLMSP1* (Tables S10–S11). For each module type observed during sequence alignment, we selected a representative sequence defined as the most frequently occurring variant within that group. These representative sequences were then used as BLAST queries to retrieve similar genotypes from public databases (GenBank IDs listed in Table S12).

The final dataset consisted of all high-confidence sequences retained after manual curation. After alignment, modular segmentation was manually defined based on recurring conserved and variable blocks. Each module’s sequence was extracted and tabulated for reference (Table S13).

### Population genetic analyses

For the most abundant haplotype (DBLMSP1-1), we performed traditional population genetic analyses. SNP data from the pf3k project [34] were used to construct DBLMSP1-1 full-length sequences across 14 countries (30 samples per country, and 60 for the Gambia, where two separate project datasets were available [1, 32]) and 450 samples in total. These datasets were reconstructed by integrating SNPs into the reference sequence using custom Perl scripts.

We then calculated nucleotide diversity (π) and Tajima’s D in DnaSP [39] with a sliding window of 100 bp and step size of 25 bp. The median-joining haplotype network was generated using Network ver10200 [3] to infer global genealogies. Population structure was analyzed using STRUCTURE v2.3.4 [37], and the optimal number of clusters (K) was evaluated via STRUCTURE HARVESTER [15]. Pairwise linkage disequilibrium (LD) was computed in DnaSP for the *R*^2^ index and plotted on heatmap graphics using the LDheatmap package [41].

In addition, amino acid mutation frequencies were tabulated for each codon in the reconstructed DBLMSP1-1 dataset (Table S14), and standard genetic diversity indices were calculated per country (Table S15).

## Results

**Figure 1:**
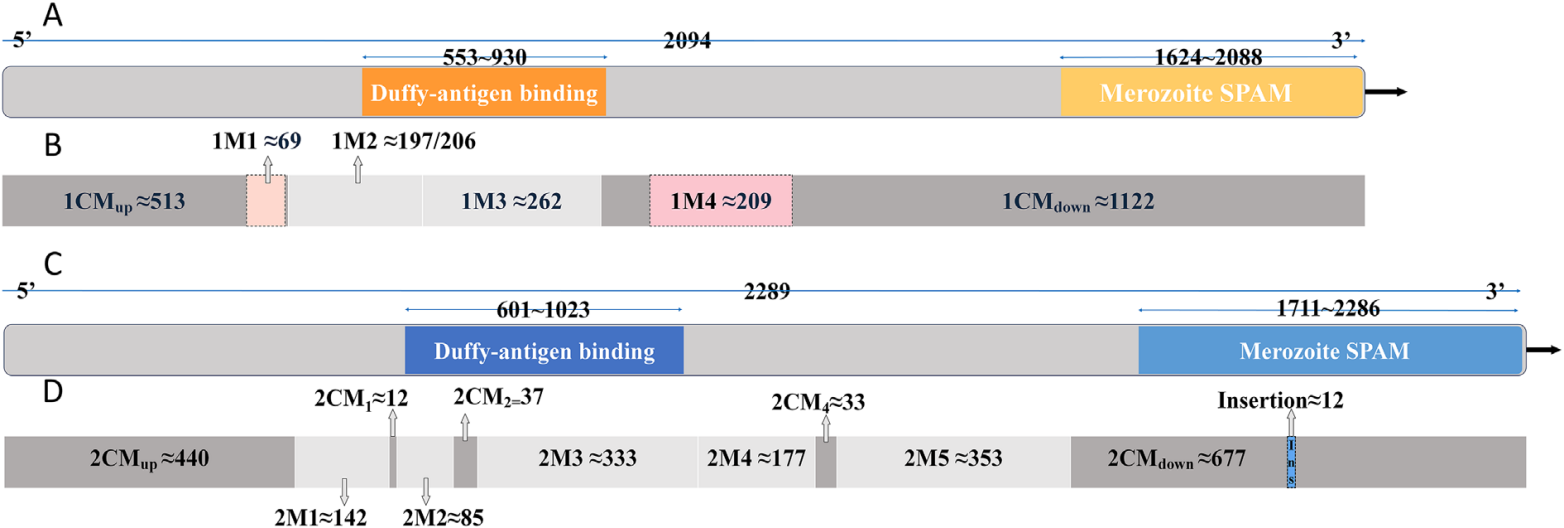
Structural comparison and modular organization of DBLMSP1 and DBLMSP2. (A) Domain structure of *DBLMSP1* (PF3D7_1035700) annotated in PlasmoDB, showing the Duffy-antigen binding domain (residues ~553–930) and the Merozoite SPAM domain (~1624–2088). (B) Modular segmentation of *DBLMSP1* based on sequence alignment. The structure includes an upstream conserved region (*1CM_up*), four variable modules (*1M1–1M4*, with *1M1* and *1M4* highlighted as highly polymorphic), and a downstream conserved region (*1CM_down*). (C) Domain structure of *DBLMSP2* (PF3D7_1036300) showing a similar organization with a DBL domain (~601–1023) and a SPAM domain (~1711–2286). (D) Modular segmentation of *DBLMSP2* includes an upstream conserved region (*2CM_up*), five variable modules (*2M1–2M5*), and a downstream conserved region (*2CM_down*). A short insertion (~12 bp) was observed downstream of the SPAM domain in some variants. Shared modules between DBLMSP1 and DBLMSP2 (*e.g.*, *1M3* and *2M5*) indicate historical recombination and intertypic exchange.

We sequenced the DBLMSP1 region from 51 *P. falciparum* isolates from the CMB region for routine population genetics analysis. However, sequence alignment revealed three distinct haplotype groups. Therefore, for each module cluster identified through multiple sequence alignment, we selected the most common variant as a representative sequence and used it to initiate a BLAST search. We then combined the BLAST alignment results and compared them with our data. We found that the differences between these haplotypes originated from some conserved or variable modules, which are widely distributed on the gene. We also found one variable segment that matched DBLMSP2, prompting us to perform an additional BLAST alignment, which retrieved a homologous sequence for DBLMSP2 and revealed a similar modular structure. Based on these findings, we propose that DBLMSP1 and DBLMSP2 possess a conserved modular structure composed of recombinant sequence fragments (Fig. 1).

**Figure 2:**
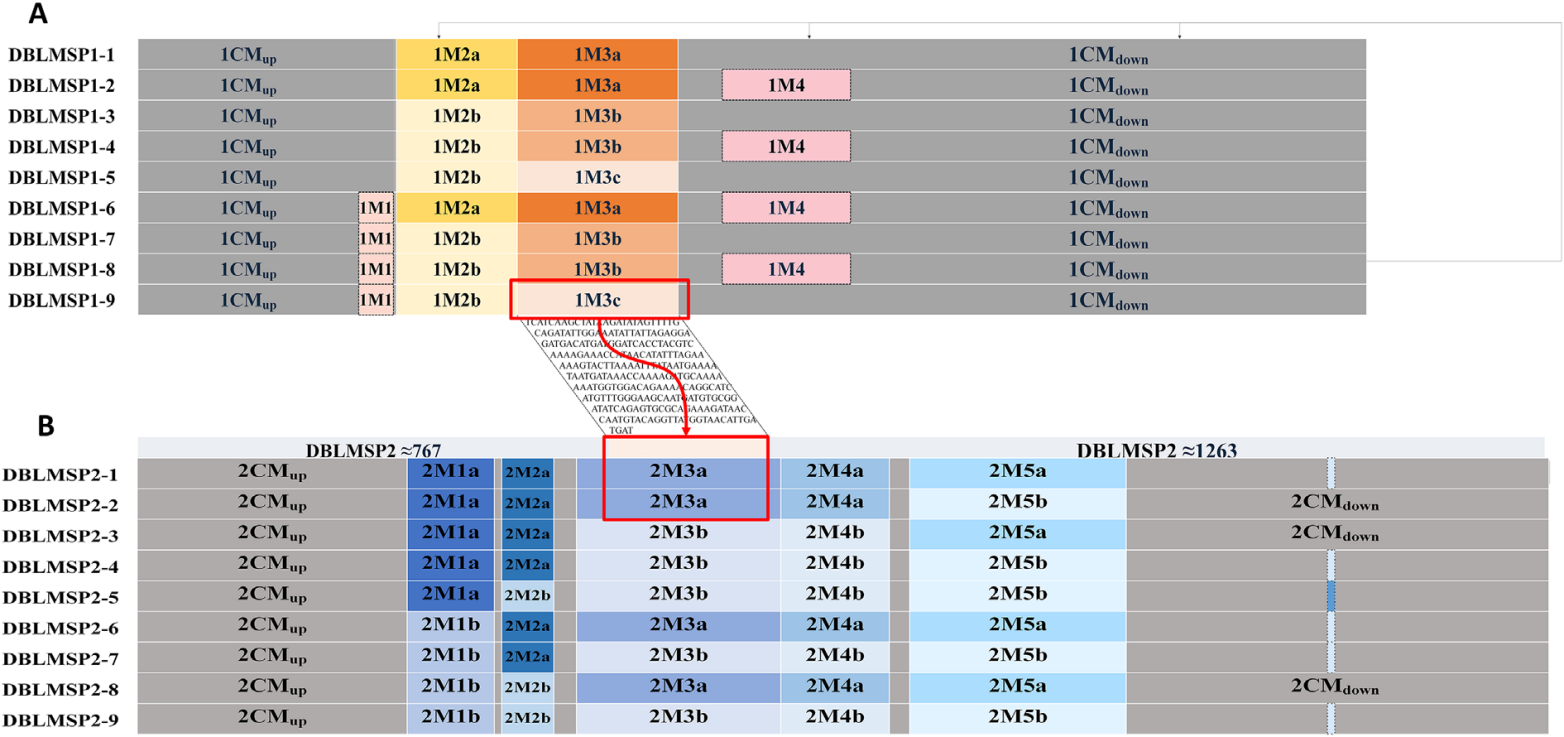
Modular configurations of DBLMSP1 and DBLMSP2 genotypes. (A) Modular composition of nine *DBLMSP1* genotypes. Each genotype comprises a conserved upstream segment (1CM_up), a variable region composed of modules 1M1–1M4, and a conserved downstream segment (1CM_down). The 1M3c module is identical in sequence to the 2M3a module of *DBLMSP2*. (B) Modular composition of nine *DBLMSP2* genotypes. Each contains conserved 2CM_up and 2CM_down regions and five variable modules (2M1–2M5). Genotypes differ by recombination and replacement among these modules. Several *DBLMSP2* genotypes share module 2M3a with *DBLMSP1* genotypes 5&9, indicating intertypic homologous recombination. A short insertion is observed in the downstream region of several *DBLMSP2* variants.

We identified nine genotypes of *DBLMSP1* and nine of *DBLMSP2*, based on unique combinations of sequence modules (Fig. 2). Each genotype consists of an invariant upstream (CM_up) and downstream (CM_down) region flanking three to four variable modules. In *DBLMSP1*, segments 1M1–1M4 account for most of the diversity, with types 1M2a/b and 1M3a–c showing distinct recombination patterns. *DBLMSP1* genotypes 6–9 harbor 1M3c, a module found to be sequence-identical to 2M3a in *DBLMSP2*, suggesting historical inter-locus recombination. Similarly, *DBLMSP2* genotypes differ primarily in their central modules (2M1–2M5), including insertions and replacements indicative of recombinational reshuffling. These shared modules indicate that *DBLMSP1* and *DBLMSP2*, although separately transcribed and located, maintain partial sequence homology via module-level exchange. Unlike the “anchoring and resolution” mechanism observed in *Anaplasma msp2* genes [8], where gene conversion events are initiated at one conserved end and resolved variably within downstream sequences [18], the *DBLMSP* sequences examined here display well-aligned module boundaries with minimal junctional ambiguity. The repeated recurrence of identical module units across distinct genotypes, without detectable hybrid junctions, supports the hypothesis of recombination through exchange of entire, pre-formed modules.

**Figure 3:**
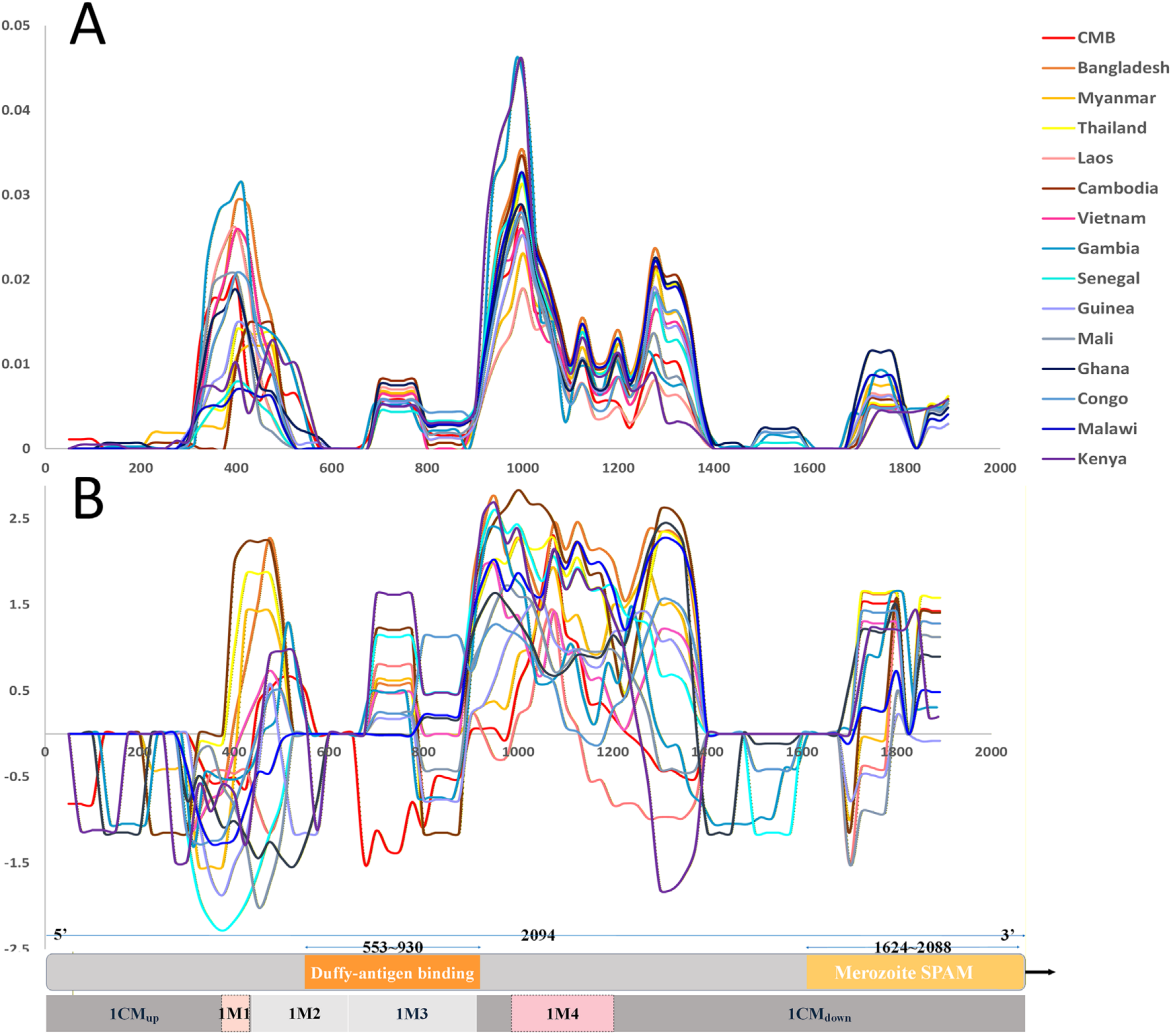
Nucleotide diversity and Tajima’s D value across the DBLMSP1-1 genotype in global *P. falciparum* populations. (A) Sliding window analysis of nucleotide diversity (π) for DBLMSP1-1 across samples from 14 countries. Diversity is lowest in the Duffy-binding domain (~553–930 bp) and SPAM domain (~1624–2088 bp), and highest within the central modular region. (B) Tajima’s D shows regional variation: values near zero within the receptor-binding domain indicate neutrality or purifying selection, whereas surrounding modules exhibit elevated D values, positive in Asian populations but negative in African populations, suggesting differences in selective pressure. The modular map below corresponds to the aligned sequence scale, showing conserved regions (gray), hypervariable modules (A–E), and structural domains.

**Figure 4:**
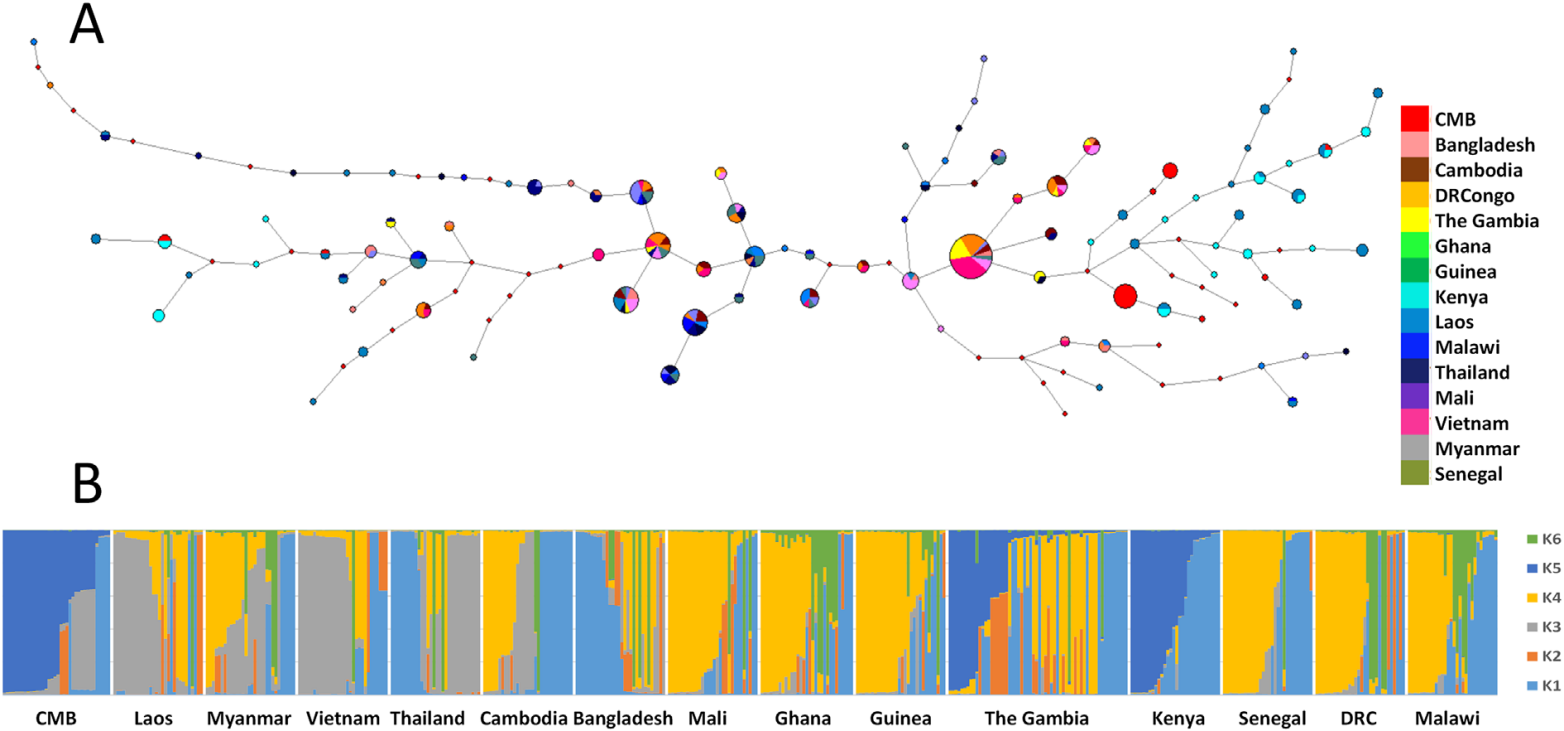
Haplotype network and population structure of DBLMSP1-1 across *P. falciparum* populations. (A) Median-joining haplotype network constructed from DBLMSP1-1 sequences. Each node represents a unique haplotype, with node size proportional to sample count and pie chart colors indicating country of origin. Two major clusters are observed, with no strong geographic partitioning. (B) STRUCTURE analysis (*K* = 6) reveals admixture among populations, with all regions showing combinations of multiple inferred clusters. Asian populations are consistently associated with cluster K3, while other clusters are broadly shared across African regions.

We downloaded the *pf3k* sequencing data, which included VCF information from 14 countries. For each country, we randomly selected 30 samples and analyzed the nucleotide diversity and Tajima’s D value in the *DBLMSP1-1* sequence alignment (Fig. 3). The results showed lower overall diversity and relatively neutral Tajima’s D values within the Duffy binding domain and SPAM domain regions, while elevated D values were observed in segments flanking the DBL domain. Haplotype network analysis revealed moderate diversity within *DBLMSP1-1*, and this dominant genotype did not show a clear geographic structure (Fig. 4A). Similarly, STRUCTURE analysis showed a widely shared genetic background, with the K3 cluster being more common in Asian populations (Fig. 4B).

**Figure 5:**
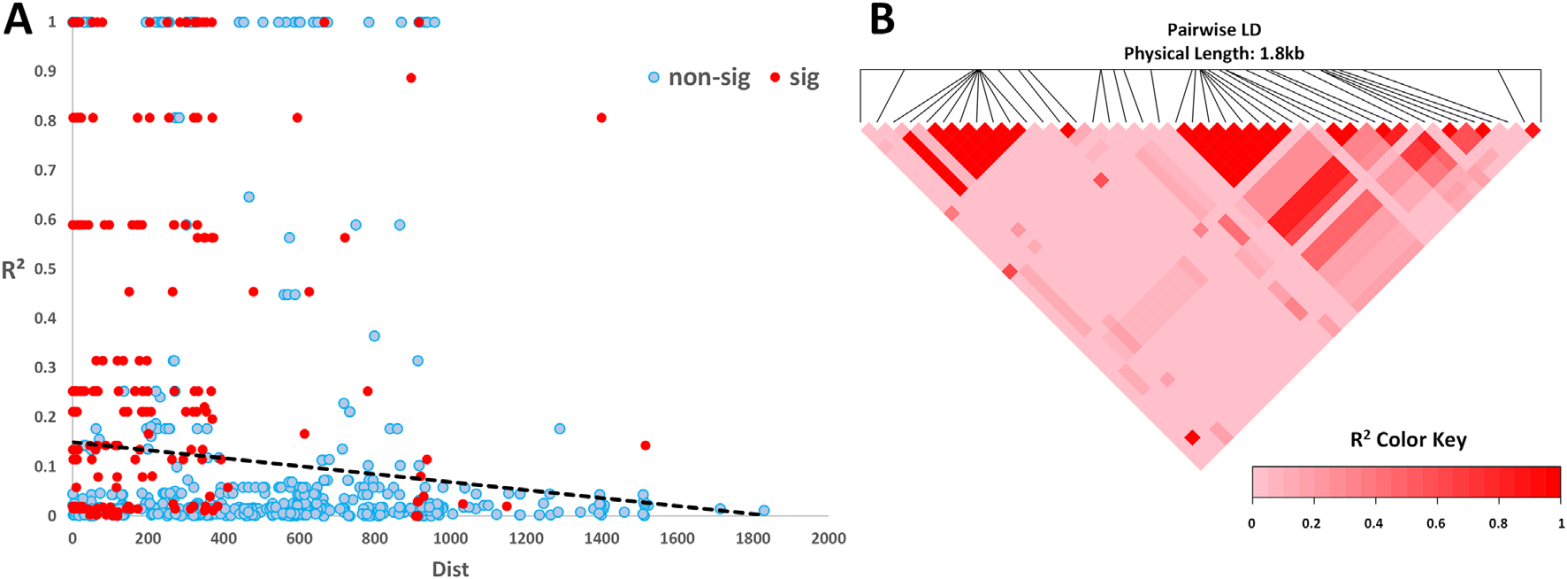
Linkage disequilibrium analysis of the DBLMSP1-1 genotype. (A) Scatter plot of pairwise LD (R^2^) against physical distance between SNPs. Significant LD values (*p* < 0.05) are shown in red; nonsignificant in blue. Although LD generally decays with distance, long-range LD blocks are evident within the ~1.8 kb region. (B) LD heatmap of pairwise SNP correlations across the same region. Several distinct LD blocks are observed, suggesting that recombination may preferentially occur between rather than within modular segments. Alternatively, strong LD could reflect selective retention of functionally compatible haplotypes, shaped by fitness constraints rather than solely by recombination suppression.

The *DBLMSP1-1* genotype exhibits significant homogeneity, and we observed several long-range linkage disequilibrium regions within this genotype (Fig. 5). These LD regions closely overlap with module boundaries, indicating that gene recombination is constrained by structural features rather than ordinary selection pressure. This modular LD pattern differs from our previous explanation that long-range LDs were entirely attributed to equilibrium selection, suggesting that modular recombination can also influence gene structure.

## Discussion

Our findings suggest that the extensive polymorphism observed in *DBLMSP1* and *DBLMSP2* is not primarily the result of diffuse point mutations, but instead appears to be concentrated within a set of discrete, recombinable sequence modules. In both genes, the modular segments – particularly 1M2 through 1M4 and their counterparts in *DBLMSP2* (2M1 to 2M5) – seem to account for most of the observed genetic and structural diversity. These regions showed elevated nucleotide diversity and Tajima’s D values, while key functional domains such as the predicted receptor-binding cleft and the C-terminal SPAM domain remained highly conserved [24]. This modular organization provides another mechanistic explanation for the antigenic variability widely reported in earlier studies.

We believe this pattern represents a form of modular intertypic homologous recombination, a well-characterized mechanism in many viruses. In RNA viruses, including *coronaviruses* [35], *enteroviruses* [33], and *retroviruses* such as HIV [9], sequence diversity often arises not from incremental base substitutions but from the exchange of large, functionally cohesive modules between related strains or serotypes. This strategy enables the rapid generation of new antigenicity while preserving key structural elements. A similar modular structure has been described in the *P. falciparum var* gene family, where DBL domains are also assembled from semi-conserved blocks that recombine across genes to maximize antigenic diversity [28, 38]. Our results extend this model to the *DBLMSP* family, which had not previously been analyzed under a modular framework. Modules encoding highly immunogenic or structurally flexible regions recombine among alleles, exhibiting high equivalence selection, while a few conserved domains (such as the DBL cleft and SPAM motif) appear to be evolutionarily constrained, possibly reflecting functional conservation. While this may not be the first instance of modular recombination in *Plasmodium*, to our knowledge, it is the first clear application of such a framework to the *DBLMSP* family. Further experimental validation will be needed to fully establish the mechanistic underpinnings of these rearrangements.

Modular recombination within DBL domains has previously been documented in *P. falciparum var* genes, where domain cassettes recombine to generate antigenic diversity [16, 26]. While this pattern is well characterized in var-type PfEMP1 proteins, it has not been systematically described in DBLMSP-family surface antigens. Our findings extend this modular paradigm to *DBLMSP1/2* and demonstrate its relevance beyond the var family. Despite significant sequence differences, previous studies have shown that the *DBLMSP1* and *DBLMSP2* alleles retain similar erythrocyte-binding capabilities [13]. Our modular hypothesis helps explain the paradox between this functional consistency and its high polymorphism. Here, core binding functions are maintained by structurally conserved modules (*e.g.*, Cleft regions), while immune escape is facilitated by variations in peripheral non-essential segments. This balance between conservation and variability may reflect a common evolutionary optimization strategy in *Plasmodium* surface antigens: the critical functions are protected from alteration, while surrounding regions diversify to evade host immune detection [20, 25]. We hope this modular hypothesis will provide a new perspective for future research on the function and variability of *Plasmodium* antigens.

*DBLMSP1* genotypes 5 and 9 all include the variable segments 1M3c, which are sequence-identical to the central segment 2M3a of *DBLMSP2.* We think this striking sequence identity, along with broader structural parallels between the two genes, raises the possibility of historical gene conversion or module-level homologous recombination. Although *DBLMSP1* and *DBLMSP2* are independently regulated and occupy distinct loci, our finding hints that they might share a modular pool that can be reshuffled under certain evolutionary pressures. Such exchangeability could carry functional or immunological implications, and we believe it merits closer attention in future studies [31].

Previous studies attributed long-range LD at the *DBLMSP2* locus to balancing selection [14, 29]. We observed similar extended LD blocks in the *DBLMSP1-1* genotype, as these sequences are modular. Long LD (non-random association between long distant SNPs) is typically thought to be due to purging selection in those regions, resulting in a lack of recombination and mutation in the population. However, in the DBLMSP1-1 genotype, we see SNP pairings with LD coinciding with the boundaries of internal modules (*e.g.*, the junctions between 1M2, 1M3, and 1M4), rather than regions clearly influenced by selection pressure. This raises the possibility that the LD patterns reflect structural constraints imposed by the modular architecture itself, rather than solely the adaptive retention of specific SNP combinations. Recombination may be less likely to occur within modules than at their boundaries, producing LD signals that mimic balancing selection. While we cannot rule out functional constraints entirely, we believe that physical boundaries between modules may play a central role in shaping LD patterns at these loci [6].

We hope that the module-based genotyping proposed in this paper can become a practical *DBLMSP* allele classification system for malaria research. This framework focuses on module composition rather than original sequence similarity, thus resolving inconsistencies in previous annotations and facilitating a more nuanced understanding of sequence diversity. More importantly, due to the conservation of modules, they may represent discrete functional or immunogenic units. Whether certain modules always correspond to major B-cell or T-cell epitopes, and whether their presence affects receptor binding efficiency or immune recognition, requires further investigation [23, 27]. This modular perspective opens promising avenues for structure-function analysis, antigen localization, and rational vaccine design.

## Data Availability

All materials and data supporting these findings are contained within the manuscript and supplementary figures and tables. The sequences have been deposited in the GenBank database under the accession numbers PX668423–PX668473 for the CMB area samples.

## Supplementary materials

*Table S1*. Type 1 Representative (CMB10) BLAST Hits. *Table S2*. Type 2 Representative (CMB42) BLAST Hits. *Table S3*. Type 3 Representative (CMB20) BLAST Hits *Table S4*. Validation of 1CM_up Module via BLAST. *Table S5*. BLAST result of 1M2a Module. *Table S6*. BLAST result of 1M2b Module. *Table S7*. BLAST result of 1M3a Module. *Table S8*. BLAST result of 1M3b Module. *Table S9*. BLAST of 1M3c Module (Shared by DBLMSP1 & DBLMSP2). *Table S10*. DBLMSP2 Reference Module BLAST. *Table S11*. Validation of 2CM_up Module via BLAST. *Table S12*. GenBank accession numbers for representative sequences corresponding to each DBLMSP1 and DBLMSP2 genotype. *Table S13*. Nucleotide sequences and positions of modular segments identified in DBLMSP1 (PF3D7_1035700) and DBLMSP2 (PF3D7_1036300). *Table S14*. Amino acid mutation frequencies in DBLMSP1-1 genotype (n = 486). *Table S15*. Genetic diversity of P. falciparum DBLMSP1-1 across different countries and regions. The supplementary material of this article is available at https://www.parasite-journal.org/10.1051/parasite/2026024/olm.
